# BoDV-1 Infection in Children and Adolescents: A Systematic Review and Meta-Analysis

**DOI:** 10.3390/pediatric15030047

**Published:** 2023-09-01

**Authors:** Matteo Riccò, Ilaria Zanella, Elia Satta, Silvia Ranzieri, Silvia Corrado, Federico Marchesi, Simona Peruzzi

**Affiliations:** 1 Occupational Health and Safety Service on the Workplace/Servizio di Prevenzione e Sicurezza Ambienti di Lavoro (SPSAL), Department of Public Health, AUSL–IRCCS di Reggio Emilia, 42122 Reggio Emilia, Italy; 2 Department of Medicine and Surgery, University of Parma, 43126 Parma, Italy; ilaria.zanella@unipr.it (I.Z.); elia.satta@unipr.it (E.S.); silvia.ranzieri@unipr.it (S.R.); federico.marchesi@unipr.it (F.M.); 3 ASST Rhodense, Dipartimento Della Donna e Area Materno-Infantile, UOC Pediatria, 20024 Garbagnate Milanese, Italy; scorrado@asst-rhodense.it; 4 Laboratorio Analisi Chimico Cliniche e Microbiologiche, Ospedale Civile di Guastalla, AUSL—IRCCS di Reggio Emilia, 42016 Guastalla, Italy; simona.peruzzi@ausl.re.it

**Keywords:** BoDV-1, Bornavirus, seroprevalence, ELISA, meningo-myeloencephalitis

## Abstract

Borna disease virus 1 (BoDV-1) can cause a severe human syndrome characterized by meningo-myeloencephalitis. The actual epidemiology of BoDV-1 remains disputed, and our study summarized prevalence data among children and adolescents (<18-year-old). Through systematic research on three databases (PubMed, EMBASE, MedRxiv), all studies, including seroprevalence rates for BoDV-1 antigens and specific antibodies, were retrieved, and their results were summarized. We identified a total of six studies for a total of 2692 subjects aged less than 18 years (351 subjects sampled for BoDV-1 antibodies and 2557 for antigens). A pooled seroprevalence of 6.09% (95% Confidence Interval [95% CI] 2.14 to 16.17) was eventually calculated for BoDV-1 targeting antibodies and 0.76% (95% CI 0.26 to 2.19) for BoDV-1 antigens. Both estimates were affected by substantial heterogeneity. Seroprevalence rates for BoDV-1 in children and adolescents suggested that a substantial circulation of the pathogen does occur, and as infants and adolescents have relatively scarce opportunities for being exposed to hosts and animal reservoirs, the potential role of unknown vectors cannot be ruled out.

## 1. Introduction

Borna disease virus 1 (BoDV-1) is a negative sense, single stranded RNA virus that belongs to the family of Bornaviridae (order Mononegavirales) alongside the Borna disease virus 2 (BoDV-2), both from genus Mammalian-1-Orthobornavirus, and the Variegated Squirrel Bornavirus 1 (VSBV-1; genus Mammalian-2-Orthobornavirus) [1,2,3,4]. The small, highly conservative genome (8.9 kilobases) is enclosed in enveloped viral particles with spherical geometry and helical capsid (70 to 130 nm in diameter for the enveloped particles, and 50 to 60 nm for the viral core) [3,4,5,6]. The viral genome is actively transcribed and replicated in the nucleus of the host cells, which is a quite uncommon feature for RNA viruses [4], and its six open-reading frames encode at least six proteins: nucleoprotein (N, or p40), phosphoprotein (P, or p24), putative matrix protein (M), type 1 membrane glycoprotein (G), and a putative polymerase (L) [7].

Since 1990, BoDV-1 has been shown as the causative agent of animal Borna disease (BoD) [5,8], a non-purulent, T lymphocyte-mediated meningo-myeloencephalitis characterized by movement disorders and behavioral abnormalities, including increased aggressivity, with a high case-fatality ratio [1,2,9,10,11,12,13]. BoD was originally described in nineteenth century as an epidemic disease of domestic mammals and livestock (mostly horses and sheep) from Southern Germany, Liechtenstein, Switzerland, and Austria [1,2,4,9,10,11,12,13]. A putative reservoir host for BoDV-1 has been recently suggested in *Crocidura leucodon* [9], the bicolored white-toother shrew, a small insectivore from the family of *Soricidae* [3,12,14,15], because of its high tolerance to BoDV-1 infection [9]. *Crocidura leucodon* likely maintains the BoDV-1 infection at local level, causing spillover events of BoDV-1 in horses and sheep through feces, urine, saliva, or even the skin [9,16]. In fact, the univocal identification of *Crocidura leucodon* as the reservoir for BoDV-1 remains elusive for several reasons. For one, the actual epidemiology of animal BoD only partially overlaps the distribution of the bicolored white-toother shrew [9,17,18]. As far as we know, *Crocidura leucodon* are distributed across central and southern Europe (with the notable exceptions of south western France, the Iberian Peninsula, and Southern Italy) eastwards to the Caspian Sea, including the countries of the Balkan peninsula, such as Poland, Ukraine, Crimea, Caucasus, Turkestan, and Iran [3,14,16], while animal BoD has been reported only in Central Europe, North America, and parts of Asia (Japan and Israel) [9,19]. Nonetheless, this apparent inconsistency may be explained by a lack of specific testing rather than by a lack of the actual circulation of the pathogen [19].

The potential for human disease has been extensively discussed in recent decades [5,20]. Since the beginning of the 1990s, high rates of seroprevalence for BoDV-1 were found in subjects characterized by chronic psychiatric conditions [5,21,22,23]. The seemingly high seropositivity rates for BoDV-1 in cases of depression or schizophrenia as well as the known neurotropism of BoDV-1 infections in animals suggest a potential link between the aforementioned psychiatric conditions and BoDV-1 [22,23,24,25,26]. However, this alleged connection has been disputed by further studies [3,5]. On the one hand, seroprevalence for BoDV-1 antibodies was identified in areas outside the known distribution of *Crocidura leucodon*, such as Japan [27,28,29,30,31,32,33,34], Thailand [35], Australia [36], Mexico [37], and the United States [25,38]. On the other hand, BoDV-1 antibodies have been described as scarcely specific [39], with a considerable suspicion that the earlier studies may have been affected by high rates of false positive results due to cross-reactivity with other pathogens [3,4] or even a laboratory contamination of samples [3,5]. However, subsequent molecular and biological analyses have dismissed the aforementioned assumptions [40,41]: even though an earlier meta-analysis of putative human BoDV-1 sequences failed to provide evidence implicating a causal role of BoDV-1 in mental illnesses [42], a recent meta-analysis has suggested that BoDV-1 infections may be associated with a substantially increased risk of schizophrenia (Odds Ratio [OR] 3.83, 95% Confidence Interval [95% CI] 1.59 to 9.20 for estimates based on Real Time Polymerase Chain Reaction [RT-PCR]) [23]. Moreover, a recent comparison of complete sequences and non-matching amino acid mutations of human isolates and shrews in the same cluster has stressed that the identification of *Crocidura leucodon* as the effective host for BoDV-1 may be more problematic than previously acknowledged, at least when dealing with human infections [40].

Since 2018, an increasing number of fatal cases of acute human encephalitis have been associated with BoDV-1 infections, hinting at the potential significance of *Bornaviridae* as the cause of acute and severe disease in humans [4,20,43,44,45,46]. For instance, in the years 2018–2019, human BoDV-1 was reported in a total of five cases of encephalitis from Germany [15,43,46], and three of them did occur in recipients of solid organ transplants (kidneys and liver) from the same donor, who had died of suspected cardiac arrest without any sign or symptoms of neurologic disease or active infectious disease [43,46]. Moreover a post-mortem RT-qPCR study on 28 cases of encephalitis or encephalopathy of unknown causality from the German state of Bavaria (1995 to 2018) detected viral RNA in seven cases (12.5%) [3]. Three further cases of BoDV-1 infection were subsequently identified through active case findings among 103 episodes of encephalitis of unknown origin that were reported in Germany at the national level (2018 and 2020) [15], and by 2022, a total of 35 cases of sporadic BoDV-1 encephalitis cases, all of which were PCR-confirmed, have been notified to the German reference center (the Robert Koch Institute) [15,20,43,47], although to date, no specific clinical features of BoDV-1 meningoencephalitis have been reported. In other words, available data suggest that both the incidence and the actual number of BoDV-1 infections in humans have been reasonably underestimated [3]. Moreover, while earlier reports have suggested that human infections could only result from occasional spillover events, particularly in agricultural settings and in individuals more likely to interact with animals (i.e., veterinarians, farm workers, etc.) [15,16,20], both zoonotic versus human-to-human transmission driven by otherwise healthy carriers should be considered [12,40]. Even though two recent and large studies from Germany have suggested that the seroprevalence of BoDV-1 may be <1%, even in areas characterized by the active circulation of the pathogen [4,47,48], the actual epidemiology of BoDV-1 in the general population still remains inconclusive [2,4,14,47], particularly in pediatric-age individuals [16,30,49,50]. In this regard, the study of BoDV-1 epidemiology in children and adolescents may be of particular interest for several reasons. For one, younger age groups have less opportunities for interacting with affected animals, mostly because of their lack of occupational exposure. Moreover, studies on animal BoD [51], seroprevalence studies in children affected by neurobehavioral disorders [23], and retrospective analysis of cases of acute encephalitis that are likely associated with BoDV-1 infection all collectively hint towards distinctive features with substantial behavioral changes and a particularly severe outcome [16], with increased morbidity and increased mortality. As a consequence, the present systematic review with meta-analysis was designed to summarize and reconcile the available data seroprevalence rate for BoDV-1 in pediatric subjects.

## 2. Materials and Methods

### 2.1. Study Selection, Inclusion and Exclusion Criteria

The present study was designed according to the PRISMA statement (Prepared Items for Systematic Reviews and Meta-Analysis) [52,53], and was then recorded in the PROSPERO (Prospective Register of Systematic Reviews) database with the ID number CRD42023426361 (PRISMA Checklist is presented in Supplement Table S1).

Research concepts, as defined according to the “PICO” strategy (Patient/Population/Problem; Intervention; Control/Comparator; Outcome), are reported in Table 1.

A combination of the following keywords: (“BoDV-1” OR “Borna virus” OR “Bornavirus” OR Borna) AND (“epidemiology” OR “seroprevalence” OR “prevalence” OR “frequency” OR “occurrence”) was searched on two scientific databases (PubMed and EMBASE) and the preprint repository MedRxiv, and modified according to the peculiarities of the database that was used. No chronological restriction was applied, while the articles were restricted to the following languages: English, Italian, German, French, or Spanish (i.e., a language understood by the reviewers). Moreover, a “snowball” approach was applied, with the references of the retrieved studies accurately searched for further suitable entries.

Inclusion and exclusion criteria are summarized in Appendix A Table A1. For the aims of this review, only original research publications written in English, Italian, German, French, or Spanish, as well as cohort studies, case-control studies, and cross-sectional studies, were analyzed, while case reports and case series were excluded if they did not provide an appropriate definition of the reference population and the corresponding seroprevalence of BoDV-1.

Further exclusion criteria were:

(1) The unavailability of the full text.
(2) Retrieved articles not including estimates on healthy children and/or adolescents (i.e., individuals ages less than 18 years).
(3) Reports lacking a proper definition of the laboratory technique(s).
(4) Reports performed only on specimens other than peripheral blood.

On the contrary, estimates on healthy children and/or adolescents as a control group from the studies that focused on the occurrence of BoDV-1 infection in schizophrenic patients were included in the analyses. Moreover, we deliberately excluded from the qualitative and quantitative analysis all of the studies that were only based on the characterization of circulating immune-complexes (CIC) and that did not provide data on BoDV-1 antigens and/or antibodies. Even though BoDV-1 specific CIC were initially considered highly sensitive and specific for natural infection [54], as they were still employed in seroepidemiological studies on BoDV-1 infections [54,55,56], the deliberate exclusion of CIC-based estimates in our study was based on claims about their inappropriate specificity [3,57,58] and also on the potential scarce reliability in earlier stages of BoDV-1 infection [55,59].

According to the PRISMA guidelines, articles were initially assessed through title screening for their relevance to the subject [52,53]. Articles that were positively title screened were then screened by the content of their abstracts. If the extracts were considered consistent with the aims and design of the present review, the full texts were independently assessed by two investigators (IZ, ES) and abstracted.

### 2.2. Data Extraction

Data extracted included:

(a) Settings of the study: year, region, targeted groups.
(b) Total number of BoDV-1 cases and their demographic characteristics.
(c) Number of the reference population (i.e., adults, if available).
(d) Characteristics of the laboratory techniques that were ultimately employed, specifically stressing the antigens and/or the targeted genetic sequences.

For the aims of the present article, a seropositive positive case of BoDV-1 was defined as:

(a) Positive status for IgG-class antibodies targeting the BoDV-1 antigens (i.e., p24, p40, and others).
(b) Positive status for BoDV-1 antigens p24 and/or p40 in blood specimens.
(c) Positive status for viral RNA at the RT-qPCR test.

### 2.3. Risk of Bias Assessment

Risk of bias of the collected papers was then assessed by means of the Risk of Bias (ROB) tool from the National Toxicology Program (NTP)’s Office of Health Assessment and Translation (OHAT) [60,61]. The ROB tool is designed to ascertain a study’s design and conduct by assessing whether the credibility of the link between exposure and outcome has been either compromised of infringed, and this leads to the eventual evaluation of the internal validity of the study under assessment. This assessment is performed through the analysis of the following main sources of bias (domains): selection of participants, confounding factors, attrition/exclusion, case detection, and selective reporting. All of the aforementioned domains are individually rated from “definitely low”, “probably low”, “probably high”, to “definitely high”, while an overall rating for each study is not provided. The OHAT handbook recommends that even studies with “probably high” or “definitely high” ratings in one or more of the assessed domains should be included in the eventual body of evidence. Ratings were provided by the two reviewers, and disagreements were resolved by consensus or by input from a third investigator (MR) where the original reviewers did not reach a consensus after extensive re-analysis of their ratings.

### 2.4. Statistical Analysis

The studies included were initially summarized by means of a descriptive analysis. Crude prevalence figures as per 100 of the population were calculated by laboratory technique (antigenic prevalence, seroprevalence, and prevalence of viral RNA, where available). The meta-analysis of the retrieved studies was then performed through a random effect model. A random effect model assumes that the true effect could vary from study to study due to the differences (heterogeneity) among studies, including participants’ demography, characteristics of the assessed intervention (e.g., the laboratory techniques), and so on [62]. On the contrary, a fixed effect model assumes that a certain true effect size underlies all of the studies that are included in the meta-analysis, and, also, that the differences in the observed effects are only due to sampling errors [62,63]. The random effect model was therefore prioritized over the fixed effect model in order to cope with the presumptive heterogenous design of retrieved studies [62,63,64].

Inconsistency (i.e., the variability across studies for a particular comparison) between the included studies was estimated by calculation of I^2^ statistic (i.e., the percentage of variation across studies that is due to heterogeneity rather than chance) [65]. In this study, I^2^ values were categorized as follows: 0 to 25% low heterogeneity; 26% to 50% moderate heterogeneity; and ≥50% substantial heterogeneity. Publication bias, or the selective publication of research studies that were based on their results, was assessed through the calculation and the subsequent analysis of contour-enhanced funnel plots, while small study bias (i.e., the asymmetric distribution of effect sizes in function of study precision) was evaluated by means of radial plots. An Egger test was performed in order to assess the potential publication bias through the asymmetry of funnel plots.

Screening of retrieved articles was performed on Mendeley Reference Manager (version 2.97.0; Mendeley Ltd.; New York, NY, USA). All calculations were performed in R (version 4.3.0) [66] and RStudio (version 2023.03.0; RStudio, PBC; Boston, MA, USA) software by means of the meta package (version 6.2-1), which is a package that provides standard methods for meta-analysis.

## 3. Results

### 3.1. Summary of Retrieved Studies

**Figure 1:**
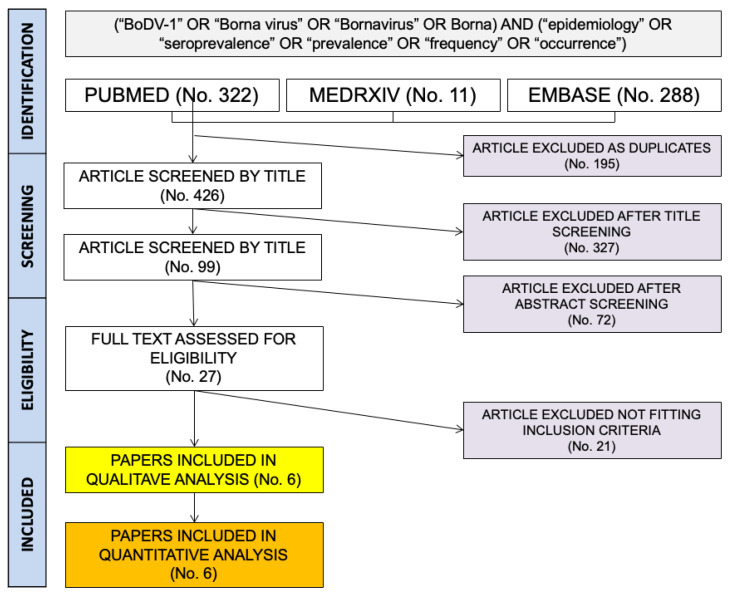
Flow chart for included studies.

Literature search results are summarized in Figure 1. More precisely, a total of 621 entries (that is: 322 from PubMed; 11 from MedRxiv; 288 from EMBASE) were identified. A total of 196 articles were removed as duplicates (31.6%), and of the remaining 426 records, 327 were removed after title screening (52.7% of the original pool) and 72 further entries were removed after the abstract screening (11.6%). A total of 27 entries were then reviewed by their full-text (4.3%), and of them, 21 were eventually excluded from both qualitative and quantitative analysis as not fitting the inclusion criteria (3.4%), while a total of 6 papers were included in the present meta-analysis (1.0% of the initial sample; Table 2).

Even though no backward chronological restriction was established, studies fulfilling the inclusion criteria were all published between 2008 and 2020, and they reported on a total of 14 estimates for 2692 individuals [49,50,51,67,68,69]. While the studies from Patti et al. [68,69] and Scholbach and Bode [51] did report on prevalence rates in an otherwise healthy population, Özcan et al. [50] and Yildirim et al. [49] included healthy children as the control for studies assessing BoDV-1 seroprevalence in selected neuropsychiatric conditions, including autism spectrum disorder.

Of the aforementioned studies, four reported on the seroprevalence for anti-BoDV-1 immunoglobulins [49,50,51,67]. All of these were performed by means of enzyme-linked immunoassay (ELISA), but significant heterogeneities were identified. For instance, the studies from Donfrancesco et al. [67], Patti et al. [68,69], and Scholbach and Bode [51] all followed the algorithm for serological diagnosis that was initially established by Bode et al. [21,40,54], with a triple ELISA that uses antibody-stabilized BoDV-1 monoclonal antibodies in order to ascertain CIC, and positive sera were then tested for antigens and negative sera for antibodies. All of the aforementioned studies employed homemade ELISA directly provided by Bode et al. [54], which specifically targeted viral protein p24 and p40, estimating the seroprevalence of both antibodies and viral antigens. The study from Özcan et al. also relied on a homemade ELISA [50,70], which was obtained through a different strategy, and which only targeted p40 antigen. On the contrary, in the study by Yildirim et al. [49], a commercial kit (Abbexa) was applied using precoated polyclonal anti-BoDV-1 IgG to detect antigens. Nothing was provided about the test protocol and the targeted antigens. No RT-qPCR based studies on healthy children and/or adolescents were ultimately retrieved.

Seroprevalence rates for BoDV-1 targeting immunoglobulins were calculated on a total of 351 subjects (13.0% of the total sample), ranging between 1.7% [51] and 25.8% [49], with a crude estimate of 21 positive cases out of 351 samples (6.0%). The prevalence rate of viral antigens was calculated on a far larger population: 2547 children and/or adolescents (94.6% of the total sample). In 216 children and adolescents, prevalence rates were reported for both BoDV-1 antigens and reactive immunoglobulins. Prevalence rates for BoDV-1 immunoglobulins ranged between no cases [67] and 20.3% [49], with a total of 44 cases (crude rate of 1.7%). Prevalence of CIC was provided by four studies and a total of 12 estimates [51,67,68,69], and the corresponding summary is provided as Appendix A Table A2.

Interestingly, half of the included studies had been performed in Italy [67,68,69], and one of them reported on the seroprevalence of BoDV-1 antigens from a total nine estimates [69], including 90.5% of all of the samples. Two further studies did report on Turkish children [49,50] and both of them included only estimates on BoDV-1 antibodies. The sixth study, meanwhile, was based in Germany, and it reported on both antigen and antibody seropositive status [51].

Focusing on the age groups, eight estimates from the studies of Donfrancesco et al. [67], Patti et al. [69], Özcan et al. [50], and Yildirim et al. [49] reported on children aged 0 to 14 years of age, while Scholbach and Bode described prevalence rates in healthy newborns [51]. Interestingly, the large study of Patti et al. [69] did also include one estimate from the Italian region of Umbria (i.e., 18 individuals), with cases aged 1 to 18 years of age, as well as the reports from the regions of Sicily (258 subjects), Sardinia (110 subjects), and Latium (116 subjects) that encompassed both children and adolescents (age range: 0 to 18 years), and a report from the region of Apulia that only included subjects aged 4 to 18 years.

### 3.2. Risk of Bias Assessment

**Figure 2:**
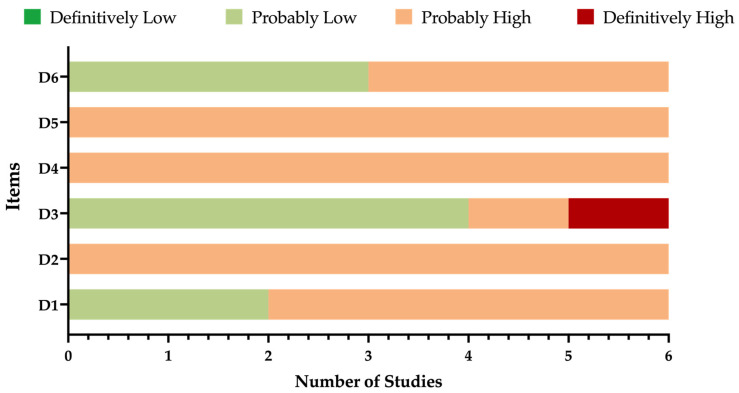
Summary of risk of bias assessment according to the National Toxicology Program (NTP)’s Office of Health Assessment and Translation (OHAT) handbook and respective Risk of Bias (ROB) tool [60,61].

A detailed description of the ROS assessment has been summarized in Table 3 and Figure 2. Briefly, the overall quality of the studies collected and summarized in the present review was mostly unsatisfying, as substantial shortcomings affected nearly all of the assessed domains. For instance, in all of the studies, the sample was recruited in terms that were quite vague, particularly in Patti et al. [68,69] and Donfrancesco et al. [67] The lack of a proper recruitment strategy led to similar uncertainties in terms of the actual potential exposure to the pathogen, which was not addressed in any of the six studies. Moreover, four of the studies (including 2557 out of 2692 potential samples, i.e., 95.0% of the total) [51,67,68,69] were published as proceedings of a conference that was conducted in 2008 [71]. Finally, even though all of the included studies were based on the ELISA technique, four of them relied on the triple testing strategy that was envisaged by the Robert Koch Institute, and they were performed by means of specifically designed monoclonal antibodies [51,67,68,69]. On the contrary, Özcan et al. [50] employed monoclonal antibodies obtained through different procedures, while Yildirim et al. [49] relied on a polyclonal commercial kit, and in both cases, diagnostic performances were not clearly stated. Even though some of the claims about the reliability of sequential testing strategy were not confirmed by more recent studies, a cautious appraisal of the reports based on the original kits from the Robert Koch Institute is still advisable, especially in view of their publication as conference proceedings [5,72,73].

### 3.3. Meta-Analysis of Included Studies

**Figure 3:**
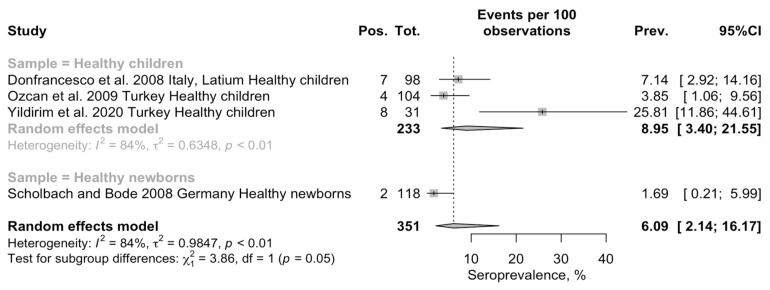
Forest plot for retrieved seroprevalence studies on antibodies targeting BoDV-1 infection in Italian Children [49,50,51,67]. A pooled seroprevalence of 6.09% (95% Confidence Interval [95% CI] 2.14 to 16.17) was calculated. Data were affected by substantial heterogeneity (τ^2^ = 0.985; I^2^ = 84.1%, 95% CI 60.0–93.7%; Q = 18.88; *p* < 0.001).

Pooled seroprevalence for BoDV-1 was estimated through a random-effect model meta-analysis in 6.09 per 100 children (95% Confidence Interval [95% CI] 2.14–16.17) (Figure 3). Overall estimates were affected by substantial heterogeneity (τ^2^ = 0.985; I^2^ = 84.1%, 95% CI 60.0–93.7%; Q = 18.88; *p* < 0.001). When considering healthy children (i.e., < 14 years of age) separately from newborns, pooled prevalence in the former group was substantially higher than among healthy newborns (8.95 per 100, 95% CI 3.40 to 21.55 vs. 1.69 per 100, 95% CI 2.14 to 16.17; chi-square = 5.811, *p* = 0.016). In fact, by assuming newborns as the reference group, the risk ratio (RR) for seropositive status among the healthy children was estimated to be 4.811, 95% CI 1.140 to 20.308. Heterogeneity was calculated only for the subgroup of the healthy children, and it remained substantial (τ^2^ = 0.635, I^2^ = 84.1, Q = 12.61).

**Figure 4:**
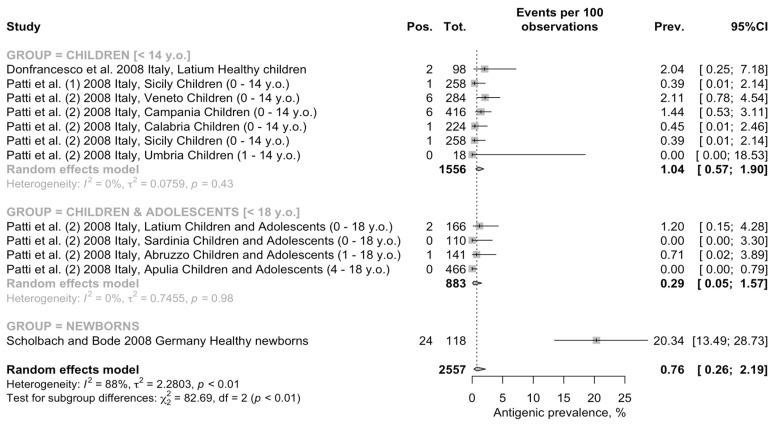
Forest plot for retrieved seroprevalence studies on BoDV-1 associated antigens in Italian Children and adolescents [51,67,68,69]. A pooled seroprevalence of 0.76% (95% Confidence Interval [95% CI] 0.26 to 2.19) was calculated. Data were affected by substantial heterogeneity (τ^2^ = 2.280; I^2^ = 87.9%, 95% CI 80.8–92.4% *p* < 0.001).

Interestingly, when focusing on the seroprevalence rate for BoDV-1 associated antigens (Figure 4), a pooled prevalence of 0.76% of people (95% CI 0.26 to 2.19) was identified. Again, the estimates were affected by substantial heterogeneity (τ^2^ =2.280; I^2^ = 87.9%, 95% CI 80.8–92.4%; Q = 91.22; *p* < 0.001).

When retrieved estimates were analyzed by age group, a quite different pattern appeared. The highest prevalence was identified among newborns (20.34 per 100 people, 95% CI 13.49 to 28.73), followed by individuals aged less than 14 years at the time of the study (1.04 per 100 people, 95% CI 0.57 to 1.90), and the lowest pooled estimate was amongst children and adolescents (i.e., <18 y.o.; 0.29 per 100 people, 95% CI 0.05 to 1.57). In the group of individuals aged less than 14 y.o. and in the group of children and adolescents, the heterogeneity was low (τ^2^ = 0.076, Q = 5.98, I^2^ = 0.0%, τ^2^ = 0.746, Q = 0.19, I^2^ = 0.0%, respectively). Moreover, assuming the prevalence rate among newborns as the reference group, RR 0.096 (95% CI 0.052 to 0.175) for children and RR 0.030 (95% CI 0.009 to 0.098) for children and adolescents were ultimately less. In fact, the seroprevalence among the subjects aged < 14 years was higher than in the samples that included both children and adolescents (age < 18 years; chi-square = 3.925, *p* = 0.048).

### 3.4. Analysis of Publication Bias

**Figure A1:**
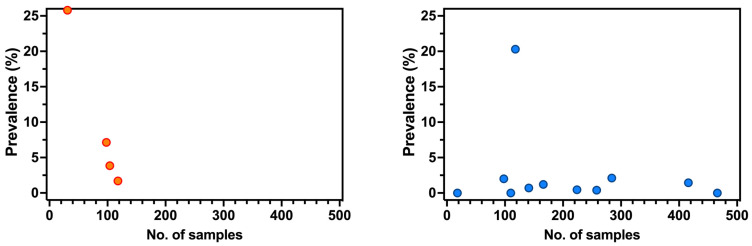
Correlation between sample size and prevalence rates for Bornavirus Disease Virus (BoDV) 1 targeting antibodies (**a**) and BoDV-1 antigens (**b**). No correlation was found in both cases (Spearman’s rho = −1.000, *p* = 0.083 for BoDV-1 antibodies, and Spearman’s ρ = 0.0247, and *p* = 0.941 for BoDV-1 antigens).

As shown in Appendix A Figure A1, no correlation was found between the overall sample size and the estimates for prevalence rates for both antibodies (Spearman’s ρ = −1.000; *p* = 0.083) and antigens (Spearman’s ρ = 0.025; *p* = 0.941).

**Figure 5:**
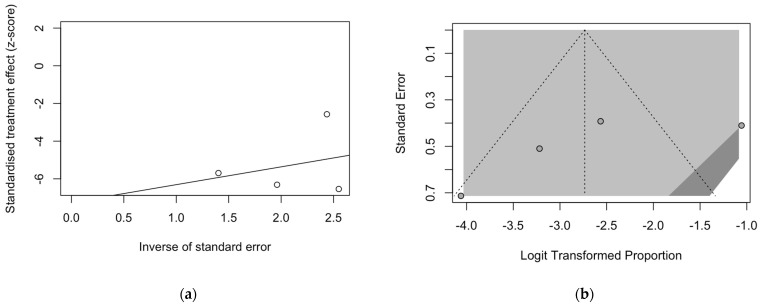
Radial plot (**a**) and funnel plot (**b**) for estimates on the prevalence of BoDV-1 antibodies. Asymmetry in both plots suggested a significant small study effect and substantial publication bias, which was rejected by Egger’s test (t = −1.42, *p* = 0.292).

**Figure 6:**
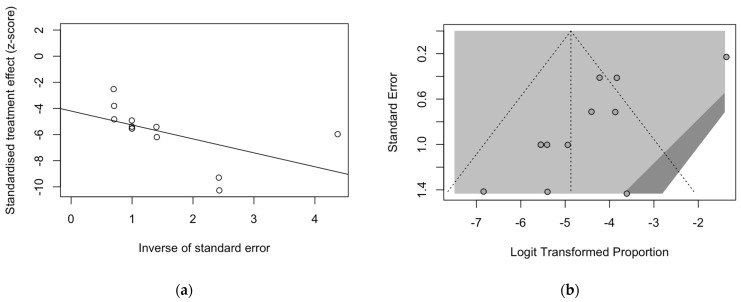
Radial plot (**a**) and funnel plot (**b**) for estimates on the prevalence of BoDV-1 antigens. Asymmetry in both plots suggested a significant small study effect and substantial publication bias. The latter was also confirmed by Egger’s test (t = −4.42, *p* = 0.001).

Analysis of publication bias and small study bias are then reported in Figure 5 and Figure 6. Even taking in account the reduced number of studies and samples that were eventually collected, inconsistent results between studies on BoDV-1 targeting antibodies (Figure 5) and BoDV-1 antigens (Figure 6) were obtained.

Regarding the prevalence of BoDV-1 targeting antibodies, a substantial effect for the small size of the samples included in the meta-analysis was hinted by the visual inspection of the radial plot (Figure 5a). On the contrary, the substantial symmetry of the corresponding funnel plot (Figure 5b) suggested the residual publication bias could be ruled out, as confirmed by Eggers’s test (t = −1.42, *p* = 0.292).

On the contrary, not only the radial plot calculated on point estimates for BoDV-1 antigens was characterized by substantial asymmetry at visual inspection (Figure 6a), as so was the corresponding funnel plot (Figure 6b), but Egger’s test hinted towards a significant publication bias (t = −4.42, *p* = 0.001).

## 4. Discussion

### 4.1. Summary of Main Findings

In the present systematic review and meta-analysis on the seroprevalence of BoDV-1 on 2692 children and adolescents from Italy, Germany, and Turkey (time frame 2008–2020), we calculated a pooled prevalence rate of 6.09% (95% CI 2.14 to 16.17) for BoDV-1 targeting antibodies and 0.76% (95% CI 0.26 to 2.19) for BoDV-1 antigens. As circulating antigens are biomarkers of ongoing infection [5,21,55,59], these results collectively suggest that BoDV-1 infections can occur in the general population, as they are detectable even in healthy infants and adolescents. Interestingly, our collective estimates on antigen and IgG anti-p24 and anti-40 are in line with a series of recent seroepidemiological studies from Germany [3,47,74]. As seroprevalence of BoDV-1 antibodies was substantially higher than that of antigens, we could also argue that the overwhelming majority of BoDV-1 infections occurs unnoticed, with cases of BoDV-1 meningoencephalitis representing nothing more than a severe but rare exception. If confirmed, our results should therefore stress the importance of identifying the individual risk factors that lead a subclinical infection into a potentially deadly disease. In turn, the reduced opportunities for occupational and environmental exposures associated with this population group would stress the importance of interhuman spreading of this pathogen.

As interesting as these results may appear, several substantial caveats should be preventively addressed that urge for a very cautious appraisal of our estimates.

### 4.2. General Interpretation in the Context of Previous Evidence

Since the isolation of BoDV-1 in 1990 [8], the true burden of human BoD has been repetitively debated [3,4,15,23,24,44]. As has recently been stressed by an editorial from Cain and Ly [75], human studies mostly performed by means of Indirect Fluorescent Antibody (IFA) previously summarized by Rott and Becht [76] have suggested a global distribution of this pathogen, with seroprevalence rates exceeding 5% or even 10% of the sampled population, and this is also the case in pediatric age groups [25]. Even more recently, reports based on the determination of CIC have hinted at very high seroprevalence rates, often 10 times higher than previous IFA testing [26,55,59]. Not coincidentally, data on CIC provided by the four studies included in our meta-analysis [51,67,68,69] point to a substantially high prevalence (53.2%; Appendix A Table A2), which would, in turn, stress the global prevalence of the pathogen and the mostly subclinical nature of BoDV-1 infections [12,25,40]. Several explanations for CIC-based estimates have been provided [21,54], including the stability of immune-complexes that result from the binding of BoDV-1 antigens with circulating antibodies. While ELISA measures circulating antigens, whose levels are considered to be transitory, CIC remain detectable for weeks or even months [21,55]. In other words, while CIC and BoDV-1 antibodies would report previous infections, with the former representing a sort of cumulative report for past interactions between the host and the pathogen, circulating antigens hint at a very recent and ongoing infection. Still, studies based on the methodology developed by Bode et al. (i.e., the sequential approach with triple ELISA) [12,21,54] have been criticized as allegedly being affected by a high risk of laboratory contamination and false positive results [5,72,73], as stressed by Rubbenstroth et al. [44]. Even though claims for potential contamination have been reasonably dismissed [40,56], more recent studies based on alternative strategies have failed to report similar prevalence rates. For instance, a recent seroloepidemiological study from Germany [47] that combined a total of 216 healthy blood donors, 280 outpatients after solid organ transplantation, and 288 serum tests from cases of suspected tick-borne disease has documented noticeable reactivity against anti-IgG ELISA in all of the assessed groups with prevalence rates in line with our results, but no indirect immunofluorescence assay-confirmed specimen was otherwise reported. As the large majority of the studies we retrieved were published in the past few decades [50,51,67,68,69], a cautious appraisal of the evidence conveyed by the reports from Patti et al. [68,69], Donfrancesco et al. [67], and Scholbach and Bode [51] is therefore mandatory [3,15,48]. Nonetheless, similar criticisms could be levelled at the reports from Yildirim et al. [49] and Özcan et al. [50]. While the former studies were performed with the very same kit that was provided by the Robert Koch Institute, only monoclonal antibodies of presumably high avidity for BoDV-1 antigens [21,54] were included, and the latter were respectively performed with monoclonal antibodies obtained through a different strategy or with commercially available polyclonal antibodies, and in both cases, corresponding diagnostic performances were not provided.

As recently summarized [4,20,45,75,77], the interest on human BoD and human infections from viruses belonging to the *Bornaviridae* family has been revamped by the study from Niller et al. [3] on cases of encephalitis of unknown origin from the German state of Bavaria, where animal BoD has been reported since the nineteenth century [9,18,19]. In this study, the authors have argued that a substantial share of the cases may find their etiology in infections sustained by species belonging to the *Bornaviridae* family, including both BoDV-1 and non-BoDV-1 species. Further studies have confirmed the association between severe human meningoencephalitis and BoDV-1 [15,16,20,43,45,47], and a more accurate definition of the actual burden of disease and potential sources of infection should be provided, as they still remain quite elusive. For example, in a recent case-control study from Germany [20] that includes 20 cases of PCR-confirmed fatal human BoDV-1 encephalitis, not a single transmission event could be found. Moreover, Tappe et al. [48] have recently stressed that seroprevalence for BoDV-1 reactive antibodies may be very low even among individuals who are potentially exposed to competent hosts, i.e., veterinarians. For instance, in their study on 736 veterinarians, only one anti-BoDV-1 IgG positive serum was identified (seroprevalence of 0.14%, 95% CI 0.00 to 0.75), while no positive case was found among 373 blood donors. In other words, both interhuman spreading of this pathogen and the role of some undefined and competent vectors cannot be easily ruled out [12] as has otherwise been indirectly suggested by our results, where estimates were performed on subjects with reduced opportunities for interaction with animals and likely hosts. In fact, the documented occurrence of BoD and BoDV-1 infection in healthcare workers [78] as well as in subjects without a clear link with any of the known intermediate hosts and reservoirs [15,20,43] demonstrate our inadequate understanding of the actual spreading pathways of these pathogens.

Some further caveats are also required. All of the studies we included in our meta-analysis did not contain accurate reporting of the living environments of the sampled children and adolescents, particularly when dealing with potential interactions with rural habitats. Elusive and problematic as it appears, the only known host for BoDV-1 is the bicolored white-toothed shrew [1,3,12,23], whose infection usually does not evolve into the severe immune-mediated encephalitis that is identified in infected horses and sheep [9,44]. However, the natural infection of this host is relatively limited compared to its actual territory [9], and even though these small insectivorous mammals are very abundant, they are also evasive. While some studies have suggested that species belonging to the genus *Crocidura* may have a very long history of interaction with humans [79], *Crocidura leucodon* can more often be found in gardens, outhouses, and farm buildings, with more limited opportunities for human interaction (and subsequent spillover) in urban settings [9,79]. As a consequence, we should expect a higher occurrence of BoDV-1 infections in the countryside as well as among individuals living in agricultural settings, as has been hinted by Pörtner et al. [20] in their case-control study. In this regard, it should be stressed that the available studies did not provide any information about the residential settings of the assessed individuals. In fact, four out of six of the retrieved studies were originally designed as conference proceedings [71], and the intention of proceedings is to function as a “precursor” for initiating more detailed primary research, thereby emphasizing the need for more studies. This may explain the lack of information in the publications, which must be acknowledged as preliminary reports.

### 4.3. Main Limitations of the Collected Results and Implications for Future Research

Despite their potential interest, our results are affected by substantial limits that should be carefully addressed. The main limits are the reduced number and low quality of the included studies as well as the fact that most of them are affected by high or a definitively high risk of bias in nearly all of the domains assessed by the ROB scale. On the one hand, four out of the six studies as well as the large majority of the sampled individuals were reported in conference proceedings, and the implicit limitations of such proceedings has been previously addressed [51,67,68,69]. More precisely, the large study of Patti et al. [69] investigated sera provided from a large serum bank, but no further details were actually provided. As it provided data on 2083 out of 2692 total samples (77.4% of total), though, its eventual impact on the pooled estimates should be acknowledged as substantial. Similarly, the study from Scholbach and Bode [51] included a total of 118 mother-newborn couples, but the authors failed to provide any detailed information about the geographical settings of the study, the actual timeframe, or the potential exposures of both the mothers and the children. On the other hand, even more recent reports are affected by similar limits in both laboratory procedures and accurate definitions of potential exposures. In the case of Yildirim et al. [49], the 31 sampled subjects in their study were otherwise healthy children that were recruited from individuals attending the Pediatric Department of Ordu University for minor complaints. Again, the authors did not provide any information about the demographics of the participants. As the eventual significance of pooled data clearly depends on the quality of primary studies [52,53,80], our estimates are also substantially affected by the limited reliability of the collected evidence. The inappropriate quality of most of the studies dealing with BoDV-1 is not radically new and has been addressed repeatedly in the last few decades [3,5,20,44,81], and the appropriate implementation of more accurately designed seroprevalence studies should therefore be undertaken [47].

Second, we identified a substantial small study effect, with smaller estimates showing higher prevalence rates than large ones. More precisely, the highest prevalence rates for BoDV-1 immunoglobulins were identified in the report from Yildirim et al. [49], which was also a smaller one (31 samples; 25.81%, 95% CI 11.86 to 44.61). Interestingly, the aforementioned report is the only one that provided seroprevalence estimates based on a polyclonal commercial kit compared to the monoclonal antibodies employed in the homemade ELISA kits from the other studies. The highest seroprevalence estimate for BoDV-1 antigens was identified in the relatively small study from Scholbach and Bode (118 samples; 20.34%, 95% CI 13.49 to 28.73) [51], but the design of the study was unable to explain whether the high rates, which suggest a recent infection of the mother-child couple, may result from the vertical transmission of the pathogen or from environmental sources. As neutralizing antibodies do not exist in the human BoDV-1 infection [4,19,75], and with maternal antibodies thus providing only limited protection of the newborn, it could be argued that the high seroprevalence for BoDV-1 antigens may reflect the first infection of an otherwise naïve individual. If otherwise documented, actual high rates of early post-natal infections would represent substantial proof of the self-limited nature of the large majority of BoDV-1 natural infections.

Third, three of the assessed estimates were provided by case-control studies on children affected by neuropsychiatric disorders [49,50,67]. As a consequence, the otherwise healthy children and adolescents included in these studies were sampled in order to be directly comparable to the cases rather than as truly representative of the pediatric age group within the general population [49,50,67,69]. Therefore, the actual representativity and generalizability of our results could be questioned.

Fourth, the studies we retrieved only reported data from three countries (Italy, Germany, and Turkey), with the large majority of the samples coming from Italy alone. While the report from Patti et al. [68,69] and from Donfrancesco et al. [67] could collectively provide some insights on the effective prevalence of BoDV-1 at the national level, the remaining studies are either unclear about the geographical representativity of their participants [51] or their participants are from a very limited area [49,50]. As a consequence, our data cannot be easily generalized at a global level, and they are reasonably affected by the local circulation of the pathogen, which adds further uncertainties to the pooled estimates.

Lastly, it should be stressed that there is no gold standard for the diagnosis of BoDV-1 infections in humans [3,4,15,47]. Several distinctive diagnostic procedures have been designed [15,20,21,40,43,48,50,54,70], and commercial kits have even been made available [49], with each one characterized by substantial advantages and disadvantages. As recently stressed by SARS-CoV-2 pandemic [82,83,84], RT-qPCR could radically improve the detection and the quantification of viral diseases in infected individuals, but its use in daily practice on BoDV-1 infections is limited [3,12,13,21,44]. In fact, RT-qPCR is a standard technique in post-mortem analysis for suspected BoDV-1 infections. Even though Bode et al. [54] recommended research into viral RNA for providing an accurate diagnosis of BoDV-1 infection, this was originally envisaged as a confirmatory test, serving as an addition but not as substitute of serology. However, all of the available diagnostic strategies were designed before the SARS-CoV-2 pandemic, and a silver lining of this global health emergency has been the renewed interest in RT-qPCR of healthcare authorities, healthcare providers, and research institutions, which, in turn, has increased diagnostic potential and performances with reduced costs and complexity, thereby ultimately making RT-qPCR a more affordable diagnostic option [85]. Future studies could thus more extensively rely on RT-qPCR testing than in previous decades. However, this option would require a more accurate definition of the natural history of BoDV-1 infection in order to identify the more suitable specimens and an appropriate testing strategy.

## 5. Conclusions

In conclusion, our study suggests that BoDV-1 infections do occur in children and adolescents, and are mostly unnoticed. As children and adolescents usually have scarce opportunities for interacting with potentially infected animals, some unknown source of infections can reasonably suspected. However, further studies will be required to better understand the actual occurrence of BoDV-1 infections, not only in children and adolescents but also in the general population. Until the proper and detailed characterization of the actual hosts, reservoirs, and competent vectors (if any) of BoDV-1 can be achieved, appropriate surveillance programs for meningoencephalitis of unknown origin should be implemented in order to properly define the epidemiology of this potentially lethal pathogen.

## Figures and Tables

**Table 1:** PICO worksheet.

Item	Definition
Population of interest	Healthy children and adolescents (<18-year-old).
Investigated result	Prevalence of biomarkers for previous exposure to BoDV-1 (i.e., BoDV-1 antigens, BoDV-1 antibodies, BoDV-1 RNA)
Control/Comparator	Healthy adults (where available).
Outcome	Seroprevalence of previous infection of BoDV-1 among in otherwise healthy children and adolescents.

**Table 2:** Summary of the series included in the qualitative and quantitative analysis on BoDV-1 infection in children and adolescents. Note: ELISA = Enzyme Linked Immuno-Assay.

Study, Year	Country	Included Groups	Laboratory Technique(s)	Prevalence of BoDV-1 Antibodies		Prevalence of BoDV-1 Antigens	
				Targeted Antigen(s)	Pos./TOT, %	Targeted Antigen(s)	Pos./TOT, %
Donfrancesco et al. [67]	Italy (Latium)	Healthy Children (0 to 14 y.o.)	ELISA, homemade	p24, p40	2/98, 2.04%	p24, p40	7/98, 7.14%
Özcan et al. [50]	Turkey (Malatya province)	Healthy Children (0 to 14 y.o.)	ELISA, homemade	Not performed	-	p40	4/104, 3.85%
Patti et al. [68]	Italy (Sicily)	Healthy Children (0 to 14 y.o.)	ELISA, homemade	p24, p40	1/258, 0.39%	p24, p40	-
Patti et al. [69]	Italy (Veneto)	Healthy Children (0 to 14 y.o.)	ELISA, homemade	p24, p40	6/284, 2.11%	p24, p40	-
	Italy (Campania)	Healthy Children (0 to 14 y.o.)	ELISA, homemade	p24, p40	6/416, 1.44%	p24, p40	-
	Italy (Calabria)	Healthy Children (0 to 14 y.o.)	ELISA, homemade	p24, p40	1/224, 0.45%	p24, p40	-
	Italy (Sicily)	Healthy Children (0 to 14 y.o.)	ELISA, homemade	p24, p40	1/258, 0.39%	p24, p40	-
	Italy (Umbria)	Healthy Children (1 to 14 y.o.)	ELISA, homemade	p24, p40	0/18, 0.00%	p24, p40	-
	Italy (Latium)	Healthy Children and Adolescents (0 to 18 y.o.)	ELISA, homemade	p24, p40	2/166, 1.20%	p24, p40	-
	Italy (Sardinia)	Healthy Children and Adolescents (0 to 18 y.o.)	ELISA, homemade	p24, p40	0/110, 0.00%	p24, p40	-
	Italy (Abruzzo)	Healthy Children and Adolescents (1 to 18 y.o.)	ELISA, homemade	p24, p40	1/141, 0.71%	p24, p40	-
	Italy (Apulia)	Healthy Children and Adolescents (4 to 18 y.o.)	ELISA, homemade	p24, p40	0/466, 0.00%	p24, p40	-
Scholbach and Bode [51]	Germany	Healthy Newborns	ELISA, homemade	p24, p40	24/118, 20.3%	p24, p40	2/118, 1.69%
Yildirim et al. [49]	Turkey (Ordu province)	Healthy Children (0–14 y.o.)	ELISA, commercial kit (Abbexa Ltd., Cambridge, UK)	Not performed	-	Not reported	8/31, 25.8%

**Table 3:** Detailed report of the Risk of Bias (ROB) estimates [60,61]. Analyses were performed according to the National Toxicology Program (NTP)’s Office of Health Assessment and Translation (OHAT) handbook and respective risk of bias (ROB) tool. Note: D1: possibility of selection bias; D2: exposure assessment; D3: outcome assessment; D4: confounding factors; D5: reporting bias; D6: other bias.

Study	Risk OF Bias					
	D1	D2	D3	D4	D5	D6
Donfrancesco et al. [67]	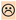	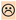	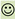	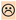	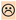	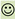
Özcan et al. [50]	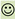	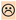	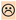	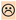	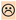	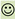
Patti et al. [68]	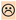	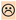	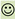	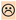	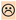	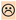
Patti et al. [69]	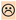	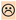	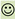	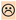	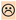	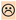
Scholbach and Bode [51]	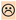	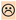	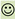	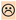	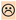	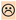
Yildirim et al. [49]	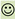	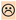	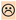 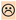	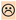	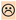	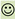

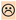

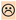
 = definitively high; 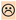 = probably high; 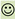 = probably low.

## Data Availability

Raw data are available from the authors of the study.

## Supplementary Materials

The following supporting information can be downloaded at: https://www.mdpi.com/article/10.3390/pediatric15030047/s1, Table S1: PRISMA 2020 Checklist.

## Appendix A

**Table A1:** Inclusion and exclusion criteria for retrieved studies. Note: ELISA = enzyme-linked immunosorbent assay; RT-PCR = real-time polymerase chain reaction; BoDV-1 = Borna Disease Virus 1.

Characteristics	Inclusion Criteria	Exclusion Criteria
Language	English, Italian, German, French, Spanish.	All other languages.
Design	Original research: cohort studies, case-control studies, and cross-sectional studies, both retrospective and prospective.	Reviews and systematic reviews. Case report and case series if unable to provide an appropriate definition of the reference population and corresponding seroprevalence of BoDV-1.
Full text	Full text available to the authors either directly or through inter-librarian loan.	Not available or results only reported by secondary studies.
Characteristics of the study population	Age ≤ 18 years	Age > 18 years or not defined.
	Healthy children and adolescents, either as the targeted population group or as a control group	Children of unknown health status or affected by neurological or psychiatric disorders.
Laboratory techniques	Accurately defined ELISA. Prevalence of circulating antibodies/antigens/RNA.	Not defined. Circulating Immune-Complexes only.
	Performed on peripheral blood.	Prevalence estimates only derived from specimens other than peripheral blood.

**Table A2:** Prevalence of circulating immune complexes (CIC) provided in collected studies.

Study, Year	Region	Total Samples	No. of Positive Samples, %
Scholbach and Bode [51]	Germany	118	27, 22.9%
Donfrancesco et al. [67]	Italy, Latium	98	50, 51.0%
Patti et al. [68]	Italy, Sicily	258	130, 50.4%
Patti et. al. [69]	Italy, Veneto	284	210, 73.9%
	Italy, Campania	416	237, 57.0%
	Italy, Calabria	224	135, 60.3%
	Italy, Sicily	258	118, 45.7%
	Italy, Umbria	18	10, 55.6%
	Italy, Latium	166	132, 79.5%
	Italy, Sardinia	110	36, 32.7%
	Italy, Abruzzo	141	79, 56.0%
	Italy, Apulia	466	231, 49.6%
Total	-	2557	1395, 54.6%
Pooled	τ^2^ = 0.398 I^2^ = 93.0% Q = 157.1 *p* < 0.001		53.2% (95% CI 43.9 to 62.3)
